# Assessing hepatitis C self-testing within differentiated care models in Cameroon: Feasibility, acceptability, and linkage to care for key and priority populations

**DOI:** 10.1371/journal.pgph.0005423

**Published:** 2025-12-15

**Authors:** Hedgar Plessy Mboussam, Stéphane Brice Seukam Kouenkap, Gutenberg Tchikangni, Idriss Tchamy, Annie Michelle Mabally, Juatio Alexis Saamene, Justin Ndié, Rose Armelle Adah, Georges Alain Etoundi Mballa, Aristide Abah Abah, Mauritius Mbella, Anne Zoung-Kanyi Bissek, Serges Clotaire Billong, Bernhard Kerschberger, Yasmin Dunkley, Mathurin Kowo, Cheryl Case Johnson, Sahar Bajis, Karin Hatzold

**Affiliations:** 1 Association Camerounaise pour le Marketing Social (ACMS), Yaoundé, Cameroon; 2 Université de Yaoundé, Yaoundé, Cameroon; 3 DROS-MINSANTE, Yaoundé, Cameroon; 4 Population Services International, Cape Town, South Africa; 5 London School of Hygiene and Tropical Medicine, London, United Kingdom; 6 Centre Hospitalier Universitaire Yaoundé, Yaoundé, Cameroon,; 7 The Department of Global HIV, Hepatitis and Sexually Transmitted Infections Programmes (HHS), World Health Organisation, Geneva, Switzerland; Dalhousie University, CANADA

## Abstract

Hepatitis C virus (HCV) infection remains a global public health concern, with limited care coverage in resource-limited settings. HCV self-testing (HCVST) offers a potential strategy to expand screening, diagnosis, and treatment. This study evaluated the feasibility and acceptability of HCVST among at-risk populations in Cameroon. Between 26-06-2023 and 01-03-2024, adults (≥21 years) were recruited through four HCVST service models targeting specific populations: people living with HIV at antiretroviral therapy clinics, men who have sex with men and people who inject drugs at drop-in-centers, and adults ≥45 years attending chronic disease clinics. Participants were randomly assigned either blood-based or oral-fluid self-test kits, with the option to test on-site or off-site, with or without assistance. Reactive results were linked to confirmatory testing and HCV treatment. Descriptive and multivariable analyses assessed acceptability and feasibility outcomes of HCVST. Of 2,653 clients offered HCVST, 99.7% (n = 2,644) accepted, 97.7% tested on-site, and 80.9% performed unassisted self-testing. Most (91.7%) found HCVST easy to use, citing rapid results (60.4%), simplicity (45.8%), and confidentiality (23.1%). Nearly all (98.7%) would recommend HCVST, with 45.9% favoring unassisted home-testing. Satisfaction with HCVST varied by care model, increased with higher HCV knowledge (aOR 1.07, 1.04-1.11), and decreased for retired clients (aOR 0.65, 0.44-0.96), blood-based tests (aOR 0.59, 0.50-0.70) and assisted testing (aOR 0.32, 0.25-0.40). Difficulties in result interpretation were rare (3.1%) but higher with off-site testing (aRR 3.76, 1.69-8.36). The HCVST seroprevalence was 4.4% (n = 117), highest at chronic disease clinics (12.2%). Among 117 clients with reactive results, 93.2% linked to confirmatory testing, 72 were treatment-eligible, and 71 (98.6%) initiated therapy, with 74.6% achieving cure. Men had greater attrition along the care cascade (aOR: 3.77, 1.10-12.91). HCVST was highly acceptable and feasible, increasing testing uptake and care engagement in Cameroon. Findings can guide rollout through differentiated, population-specific delivery models.

## Introduction

Hepatitis C virus (HCV) remains a significant global public health challenge, with an estimated 50 million people worldwide living with the HCV [1]. Untreated infection can result in severe liver-related complications (e.g., cirrhosis, liver failure, and hepatocellular carcinoma), causing approximately 240,000 deaths annually in 2022 [1]. Many cases remain asymptomatic for years, contributing to low diagnosis and delayed treatment [2,3]. Timely detection and therapy with well-tolerated direct-acting antivirals (DAAs), which have revolutionized HCV care with cure efficacy exceeding 95%, are essential to preventing long-term health consequences and achieving global elimination targets [1,4,5].

The African region is particularly affected, with an estimated 8 million people living with HCV in 2022, of whom only 13% were diagnosed and 3% received treatment [1]. Limited access to testing remains a major barrier, driven by under-resourced healthcare systems, low awareness of viral hepatis, out-of-pocket expenditure, and limited availability of routine testing services [6,7]. Furthermore, the integration of HCV care within existing health systems is often lacking [6], particularly for high-risk and key populations, including people who inject drugs (PWID), men who have sex with men (MSM) and people living with HIV (PLHIV). Many individuals testing positive do not receive follow-up treatment assessments or access DAAs therapy [1].

The recent development of HCV self-testing (HCVST) kits offers new opportunities to enhance HCV care delivery [8–11]. Drawing from lessons in HIV self-testing [11–13], HCVST offers the potential for greater accessibility and convenience by allowing individuals to privately determine their HCV status [8–11], therefore reducing dependance on clinical visits. By leveraging community-based and secondary distribution models, HCVST may even extend its reach into high-risk networks, thus enhancing detection among populations underserved by traditional healthcare systems. However, HCVST is not yet available within the public health sector although it has been recommended by WHO as a complementary approach to existing testing services [14]. To our knowledge, experience from low-resource settings remains limited to a few small-scale studies which reported favourable usability and acceptability among general and key populations [15–18], and modelling suggesting that HCVST can be cost-effective in high-prevalence contexts by increasing diagnosis, treatment uptake, and cure [19].

Cameroon has among the highest HCV seroprevalence in Africa, ranging from 3.6% in the general population to 12.2% in high-risk groups such as healthcare workers and individuals with comorbidities [20,21]. The epidemic is genetically diverse, varies by age and geography, and is compounded by limited access to affordable testing and care [7,21–23]. Assessing the acceptability and feasibility of new interventions such as HCVST is essential to determine whether self-testing can be effectively used in this context and integrated into existing health systems, particularly among underserved populations. Therefore, we evaluated the acceptability and feasibility of HCVST in Cameroon using differentiated care models based on facility- and community-based delivery approaches that were tailored to the access needs and vulnerabilities of specific at-risk populations.

## Materials and methods

### Study design and target populations

This prospective cohort study evaluated the acceptability and feasibility of blood-based and oral-fluid-based HCVST among at-risk and key populations in Yaoundé and the Central Region of Cameroon between 26-06-2023 and 01-03-2024. A secondary objective was to assess progression along the HCV care cascade, from a reactive self-test to cure.

Adults (≥21 years) from five groups were enrolled:

- PLHIV attending three HIV treatment clinics (ARTCs);
- MSM at a key population drop-in center (MSM-DIC);
- PWID at a PWID-specific drop-in center (PWID-DIC) that served both current and past injecting drug users;
- Secondary distribution to sexual or drug-injecting contacts of HCV-positive index cases among MSM and PWID;
- Individuals ≥45 years presenting at outpatient chronic disease clinics (CDCs).

**Fig 1:**
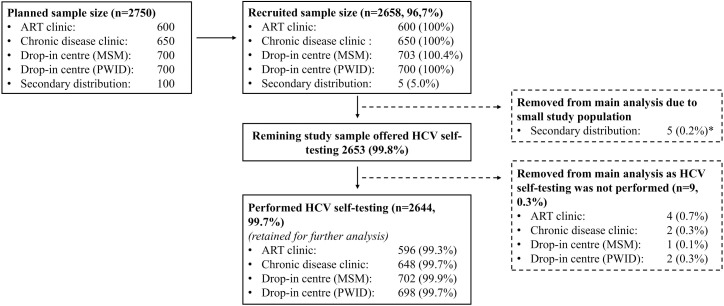
Study flow chart. HCV, hepatitis C virus; MSM, men who have sex with men; PWID, people who inject drugs; n, number. Secondary distribution has been removed from the main analysis due to its small sample of participants. A description of secondary distribution is, however, provided in additional analysis.

Populations were selected based on high documented HCV prevalence (e.g., 55.9% among individuals >60 years) and known risk factors (e.g., HIV co-infection, behavioral exposures). The study aimed to recruit 2,750 participants (Fig 1).

### Implementation procedures

Self-test kits were distributed through two main approaches:

1. Facility-based distribution at three referral hospitals (Central, General, and Yaoundé University Hospitals), comprising the entry points ARTCs and CDCs;
2. Community-based distribution through two separate MSM or PWID focused DICs, including secondary distribution to their sexual or needle-sharing contacts.

Hereafter, care models refers to the five differentiated distribution models (ARTC, CDC, DIC-MSM, DIC-PWID, secondary distribution) with a description of characteristics provided in Table 1.

**Table 1:** Characteristics of HCV self-testing care models.

Distribution approach	Facility-based^a^	Facility-based^a^	Community-based	Community-based	Community-based
**Entry point/ care model**	HIV clinic^b^	Chronic care clinic^c^	Drop-in center for MSM^d^	Drop-in center for PWID^d^	Index case testing^e^
**Target population**	PLHIV	General at-risk population with comorbidities (≥45 years)	MSM	PWID	Sexual and injecting partners of MSM and PWID
**Distribution model**	Primary distribution	Primary distribution	Primary distribution	Primary distribution	Secondary distribution
**Service provider**	Public sector	Public sector	Non-governmental organizations	Non-governmental organizations	HCV positive index client^d^

HCV, hepatitis C virus; MSM, men who have sex with men; PLHIV, people living with HIV; PWID, people who inject drugs.

^a^The Central, General, and Yaoundé University Hospitals offered outpatients care at HIV clinics for antiretroviral therapy initiation and refills, and chronic disease care.

^b^These comprised patients newly diagnosed with HIV and already receiving antiretroviral therapy for HIV.

^c^These clinics primarly serve adults seeking care for non-communicable diseases such as hypertension, diabetes, asthma, and cardiovascular disease. They provide general consultations and manage a mix of walk-in clients and scheduled follow-ups.

^d^The drop-in center for MSM offered mobilization, education, HIV care, violence support, and advocacy. The drop-in center for PWID provided HIV prevention, psychosocial care, tuberculosis care, referrals, and risk counselling.

^e^Clients with confirmed active HCV infection in the drop-in centers for MSM and PWID were provided with HCV self-testing kits for distribution to their sexual and injecting partners.

Participants were consecutively recruited at facilities or mobilized in communities by peer educators. A baseline questionnaire captured socio-demographics, HCV risk factors, and HCV knowledge. Participants were randomly assigned to a blood-based (First Response HCV Card Test, Premier Medical Corps Ltd., India) or oral-fluid-based (OraQuick HCV, OraSure Technologies Inc., USA) self-test, both detecting HCV antibodies without distinguishing active from past infections. Randomisation was performed using a computer-generated list stratified by site and key population, administered centrally by the study coordination team. According to WHO Prequalification Reports [24,25], the First Response HCV Card Test and the OraQuick HCV self-test demonstrate comparable performance characteristics with both tests showing a sensitivity of 100% and specificity of 99.7%.

Instructions were provided before testing. Participants self-tested on-site (with or without assistance) or off-site, with follow-up calls to confirm results. Post-test interviews assessed user experience and linkage outcomes.

Reactive self-testers were referred to HCV referral centers for confirmatory diagnosis (provider administered rapid testing, quantitative HCV ribonucleic acid (RNA) testing, liver function assessment, clinical evaluation). Participants with detectable HCV RNA were initiated on free DAAs (Sofosbuvir/Velpatasvir) as per national guidelines, with cure confirmed by an undetectable HCV RNA 12 weeks after treatment initiation. Those with undetectable HCV RNA received counseling on harm reduction, reinfection risks, and safer practices, and were scheduled for a follow-up HCV RNA test after six months to monitor for possible reinfection.

MSM and PWID with confirmed current infection were offered up to three additional self-tests to distribute to recent sexual or injecting partners personally known and reachable to them. These secondary contacts were not directly enrolled by the study team. Index participants were encouraged to provide verbal instructions, with counselling, confirmatory testing, and study enrolment services available to secondary users who voluntarily presented at the DICs.

All study staff and peer educators received standardised training on the study protocol, HCVST procedures, and data collection tools prior to implementation. Training included practical demonstrations and use of job aids. Instruction materials were provided in French and included illustrations to support comprehension. Peer educators were additionally trained on community mobilisation and supporting self-test users.

### Outcome variables

#### Acceptability.

Acceptability was assessed through user satisfaction, confidence in the test, ease of use, and willingness to recommend or reuse HCVST. Ten questions captured perceptions across two domains: (1) the testing process (ease of following instructions, opening and handling the kit, reading results, and the overall process) and (2) testing-related qualities (ease, convenience, privacy, comfort, and trust in results). Responses were recorded on 5-point Likert scales (1 = very low, 5 = very high), with higher scores indicating greater acceptability. Terms such as ‘very easy,’ ‘very high,’ ‘very confident,’ or ‘very satisfied’ reflect these self-reported Likert-scale ratings and correspond to participants’ subjective perceptions across different aspects of the HCV self-testing experience. These acceptability indicators were developed specifically for this study, informed by comparable HIV self-testing research and relevant HCV literature, and were tailored to the local HCV testing context in Cameroon.

#### Feasibility.

Feasibility was defined as HCVST uptake, test completion, ability to self-test (with or without assistance), and reported difficulties in result interpretation. Linkage to care and treatment outcomes were also tracked as downstream effects. The HCV care cascade included confirmatory provider administered rapid-diagnostic testing, quantitative HCV RNA testing, treatment initiation, and cure, defined as an undetectable HCV RNA following therapy with DAAs. Treatment eligibility required both a positive provider-administered antibody test and a detectable HCV RNA. Attrition from HCV care was defined as failure to complete one or more steps along the care cascade following a reactive HCVST result, including confirmatory provider-administered rapid diagnostic testing, HCV RNA quantification, treatment initiation, or achieving cure.

### Data sources

Data were collected through structured, paper-based questionnaires during face-to-face interviews or telephone follow-up after HCVST use, and entered into an electronic database using SurveyCTO. A random 20% sample was checked for data consistency.

### Sample size

The primary outcome used for precision sample size estimates was uptake of HCV self-testing at point of offer (proportion of eligible clients who accepted testing). Allocations were planned pragmatically across care models based on programmatic expectations of participation. Using a conservative assumption p = 0.5 at 95% confidence, yielded ±1.9% precision for overall uptake (N = 2750), and between ±3.7–4.0% within each stratum (ARTC n = 600, CDC n = 650, MSM-DIC n = 700, PWID-DIC n = 700). Secondary distribution analyses targeted n = 100 for exploratory analyses.

### Statistical analysis

Descriptive statistics (frequencies and proportions) summarized client baseline characteristics, self-testing approaches, experiences, future recommendations, and progression along the HCV care cascade. Crude 5-point Likert scale responses were also summarised using frequencies and proportions for each response category. Differences across care models were assessed using Pearson’s chi-squared test.

Ten separate multivariable ordered logit models were constructed to examine associations between baseline factors and each testing experience outcome. Explanatory variables included care model, sociodemographic factors, recent HCV risk behaviors, HCV knowledge, and self-testing-related determinants. Proportional odds were assumed for ease of interpretation.

To assess overall HCVST experience, individual scores across the 10 experience domains were averaged and recategorized into a single 5-point Likert variable. Then, a generalized ordered logistic regression model with robust 95% confidence intervals was first fitted under the proportional odds assumption for simplicity. In a secondary analysis, the proportional odds assumption was relaxed where violations were observed (e.g., for care model and needle sharing) using the gologit2 command in Stata [26]. This approach allowed the associations of these predictors to vary across different levels of satisfaction, providing a more accurate representation when their effects were not constant across the outcome.

Self-reported difficulty in interpreting HCVST results was analyzed using a multivariable log-binomial regression model, appropriate for rare outcomes (<10%).

The HCV care cascade was described from reactive HCVST results to treatment outcomes. Supplementary analyses of cumulative attrition along the HCV care cascade were conducted using multivariable logistic regression with penalized likelihood estimation to address sparse data.

Clients in the secondary distribution model (n = 5) were excluded from the primary analysis due to small sample size; their data were analyzed separately.

All analyses were performed using Stata 17.

### Ethics

The study was approved by the National Ethics Committee of Cameroon (2023/01/1930/L/CNERSH/SP), the Population Services International Research Ethics Board (39.2022), and the WHO Research Ethics Committee (ERC.0003850). Written informed consent was obtained from all participants.

## Results

### Study enrollment

The study flow chart is presented in Fig 1. Overall, the targeted sample size was achieved across all care models, except for secondary distribution, where only 5.0% (n = 5) of the target was met. Among the 2,653 clients offered HCV self-testing, only nine declined, resulting in an overall uptake of 99.7% (n = 2,644). Self-testing uptake was consistently high across all care models: ARTC: n = 596, 99.3%; CDC: n = 648, 99.7%; MSM-DIC: n = 702, 99.9%; PWID-DIC: n = 698, 99.7%.

### Baseline characteristics

#### Socio-demographic and behavioral factors.

Baseline characteristics varied significantly across care models (n = 2,644; Table 2). Overall, 67.2% were men, 52.8% had completed secondary education, and the median age was 36 years (IQR: 25–52). Younger participants (21–29 years) predominated at DIC-MSM (80.6%) and DIC-PWID (53.6%), while older adults were more common at ARTC and CDC. Most participants were single (58.7%), particularly at DIC-MSM (91.0%) and DIC-PWID (85.1%), while marriage was more common at ARTC and CDC. Employment was highest in DIC-PWID (75.1%) and ARTC (67.3%), with students concentrated at DIC-MSM (42.6%). No sexual contact was reported by 25.1% of participants, with the highest proportion observed at the CDC (46.0%) and the lowest at the MSM-DIC (6.8%). Protected and unprotected same-sex and bisexual sexual contacts were most commonly reported at the MSM-DIC (77.9%), whereas unprotected heterosexual contact was the predominant behaviour at the CDC, reported by 47.7% of clients. Overall, 13.0% reported an STI diagnosis in the past six months, highest at DIC-MSM (20.5%), and 19.3% reported recent needle sharing, with the highest prevalence at DIC-PWID (56.2%).

**Table 2:** Baseline characteristics of clients accessing and performing HCV self-testing.

	Total	ARTC	CDC	DIC-MSM	DIC-PWID	p-value
**Age group, years**						
21-29	1001 (37.9)	61 (10.2)	0 (0.0)^b^	566 (80.6)	374 (53.6)	<0.001
30-39	466 (17.6)	108 (18.1)	0 (0.0)^c^	115 (16.4)	243 (34.8)	
40-49	415 (15.7)	182 (30.5)	147 (22.7)	15 (2.1)	71 (10.2)	
50-59	395 (14.9)	152 (25.5)	232 (35.8)	3 (0.4)	8 (1.1)	
≥60	367 (13.9)	93 (15.6)	269 (41.5)	3 (0.4)	2 (0.3)	
**Sex**						
Men	1778 (67.2)	172 (28.9)	267 (41.2)	702 (100.0)	637 (91.3)	<0.001
Women	866 (32.8)	424 (71.1)	381 (58.8)	0 (0.0)	61 (8.7)	
**Education completed**						
No education	37 (1.4)	9 (1.5)	12 (1.9)	0 (0.0)	16 (2.3)	<0.001
Primary	394 (14.9)	146 (24.5)	155 (23.9)	14 (2.0)	79 (11.3)	
Secondary	1396 (52.8)	312 (52.3)	320 (49.4)	247 (35.2)	517 (74.1)	
Tertiary	817 (30.9)	129 (21.6)	161 (24.8)	441 (62.8)	86 (12.3)	
**Employment status**						
Working	1622 (61.3)	401 (67.3)	371 (57.3)	326 (46.4)	524 (75.1)	<0.001
Not working	277 (10.5)	41 (6.9)	62 (9.6)	38 (5.4)	136 (19.5)	
Student	367 (13.9)	33 (5.5)	1 (0.2)	299 (42.6)	34 (4.9)	
Retired	207 (7.8)	43 (7.2)	162 (25.0)	2 (0.3)	0 (0.0)	
Other	171 (6.5)	78 (13.1)	52 (8.0)	37 (5.3)	4 (0.6)	
**Marital status**						
Single	1552 (58.7)	230 (38.6)	89 (13.7)	639 (91.0)	594 (85.1)	<0.001
Married	811 (30.7)	246 (41.3)	409 (63.1)	60 (8.5)	96 (13.8)	
Divorced*	281 (10.6)	120 (20.1)	150 (23.1)	3 (0.4)	8 (1.1)	
**HCV knowledge** ^**a**^ **, (median, IQR)**	3 (0-6)	4 (1-6)	3 (0-6)	5 (2-6)	0 (0-2)	<0.001
**Sexual behavior (past 6 months)** ^**b**^						
No sexual contact	663 (25.1)	209 (35.1)	298 (46.0)	48 (6.8)	108 (15.5)	<0.001
Unprotected heterosexual contact	859 (32.5)	210 (35.2)	309 (47.7)	38 (5.4)	302 (43.3)	
Protected heterosexual contact	563 (21.3)	175 (29.4)	40 (6.2)	69 (9.8)	279 (40.0)	
Unprotected same-sex contact	104 (3.9)	1 (0.2)	0 (0.0)	100 (14.2)	3 (0.4)	
Protected same-sex contact	212 (8.0)	0 (0.0)	1 (0.2)	205 (29.2)	6 (0.9)	
Unprotected bisexual contact	99 (3.7)	0 (0.0)	0 (0.0)	99 (14.1)	0 (0.0)	
Protected bisexual contact	144 (5.4)	1 (0.2)	0 (0.0)	143 (20.4)	0 (0.0)	
**STI diagnosis (past 6 months)**						
No	2300 (87.0)	545 (91.4)	617 (95.2)	558 (79.5)	580 (83.1)	<0.001
Yes	344 (13.0)	51 (8.6)	31 (4.8)	144 (20.5)	118 (16.9)	
**Sharing needles (past 6 months)**						
No	2134 (80.7)	563 (94.5)	606 (93.5)	659 (93.9)	306 (43.8)	<0.001
Yes	510 (19.3)	33 (5.5)	42 (6.5)	43 (6.1)	392 (56.2)	

ARTC, antiretroviral therapy clinic; CDC, chronic disease clinic; DIC-MSM, drop-in center for men who have sex with men; DIC-PWID, drop-in center for people who inject drugs; HCV, hepatitis C virus; HCVST, hepatitis C virus self-testing; IQR, interquartile range; STI, sexually transmitted infection.

^a^Current HCV knowledge was assessed using 8 questions. Participants who had never heard of HCV were assigned a score of 0, while others received cumulative scores based on correct answers. Scores ranged from 0 to 8, with higher scores indicating greater knowledge, and were treated as a continuous variable.

^b^Sexual behavior categories were constructed by combining reported condom use and the gender of sexual partners in the past 6 months. Participants reporting no sexual contact were classified accordingly. Among sexually active individuals, those who reported ‘never’ or ‘rarely’ using condoms were categorized as having unprotected sex, while those reporting condom use ‘often’ or ‘always’ were categorized as having protected sex. This composite variable reflects sexual behaviour but not sexual orientation or gender identity.

^c^Clients at CDC were eligible for the study only if they were aged ≥45 years.

#### Approaches to self-testing.

The type of HCVST kit used was consistent across care models, with nearly half of participants (n = 1318; 49.8%) receiving oral-fluid-based kits, ranging from 47.1% at CDC to 52.9% at PWID-DIC (Table 3). On-site testing was the predominant approach (97.7%), and most participants performed HCVST unassisted (80.9%). However, assistance was more commonly used in PWID-DIC (39.4%) compared to the overall sample (19.1%), and off-site testing was most prominent at ARTC (5.0%).

**Table 3:** HCV antibody self-testing characteristics by care model.

	Total	ARTC	CDC	DIC-MSM	DIC-PWID	p-value
**Type of HCV self-test**						
Oral	1318 (49.8)	304 (51.0)	305 (47.1)	340 (48.4)	369 (52.9)	0.143
Blood	1326 (50.2)	292 (49.0)	343 (52.9)	362 (51.6)	329 (47.1)	
**Location of HCV self-testing**						
On-site	2583 (97.7)	566 (95.0)	627 (96.8)	692 (98.6)	698 (100.0)	<0.001
Off-site	61 (2.3)	30 (5.0)	21 (3.2)	10 (1.4)	0 (0.0)	
**Level of assistance needed**						
Unassisted	2137 (80.9)	483 (81.5)	561 (86.6)	670 (95.4)	423 (60.6)	<0.001
Assisted	504 (19.1)	110 (18.5)	87 (13.4)	32 (4.6)	275 (39.4)	
**HCVST screening interpretation**						
Non-reactive result	2445 (92.5)	554 (93.0)	551 (85.0)	665 (94.7)	675 (96.7)	<0.001
Reactive result	117 (4.4)	18 (3.0)	79 (12.2)	10 (1.4)	10 (1.4)	
Test failure	66 (2.5)	22 (3.7)	8 (1.2)	27 (3.9)	9 (1.3)	
Unable to read result	14 (0.5)	2 (0.3)	10 (1.5)	0 (0)	2 (0.3)	
Unwilling to disclose result	2 (0.1)	0 (0)	0 (0)	0 (0)	2 (0.3)	

HCV, hepatitis C virus; HCVST, hepatitis C virus self-testing.

### HCVST outcomes

Overall, 4.4% (n = 117/2644) of all clients reported a reactive HCVST result (Table 3). HCV reactivity was highest at the CDC (n = 79/648, 12.2%), followed by the ARTC (n = 18/596, 3.0%), and lowest at community-level (MSM-DIC: n = 10/702, 1.4%; PWID-DIC: n = 10/698, 1.4%) (p < 0.001). Among key demographic characteristics, women were more likely to have reactive results (7.3% [n = 63/803] vs. 3.0% [n = 54/1724] for men; p < 0.001) as well as older age groups (18.5% [n = 68/299] in ≥60 years and 5.3% [n = 21/374] in 50–59 years vs. 1.2% [n = 12/989] in 21–29 years; p < 0.001).

### Acceptability

#### Overall.

**Fig 2:**
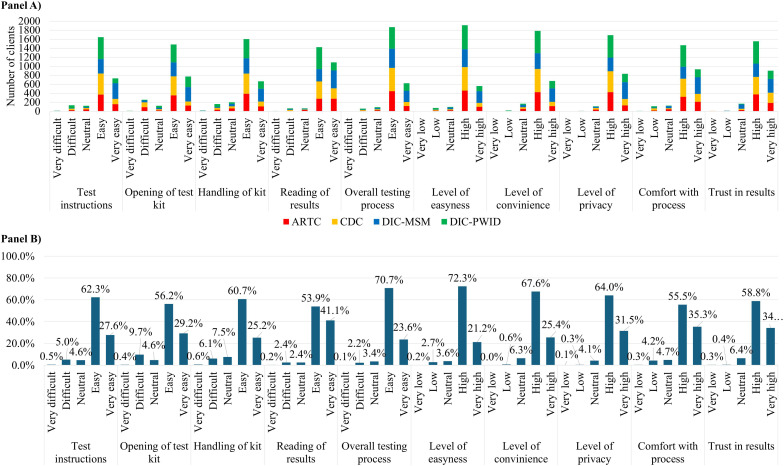
Absolute number of responses by test domain and care model (panel A) and overall proportions of satisfaction levels within domains (Panel B). ARTC, antiretroviral therapy clinic; CDC, chronic disease clinic; DIC-MSM, drop-in center for men who have sex with men; DIC-PWID, drop-in center for people who inject drugs.

Most participants rated the HCVST process as easy or very easy: following instructions (89.9%), opening the kit (85.3%), handling the kit (85.8%), reading results (95.0%), and the overall process (94.3%) (Fig 2, Panels A and B; S1 Table). Related qualities also received high or very high ratings, including ease of use (93.5%), convenience (93.0%), privacy (95.5%), comfort (90.8%), and trust in results (93.0%).

#### Domain-specific associations with HCVST experiences.

**Fig 3:**
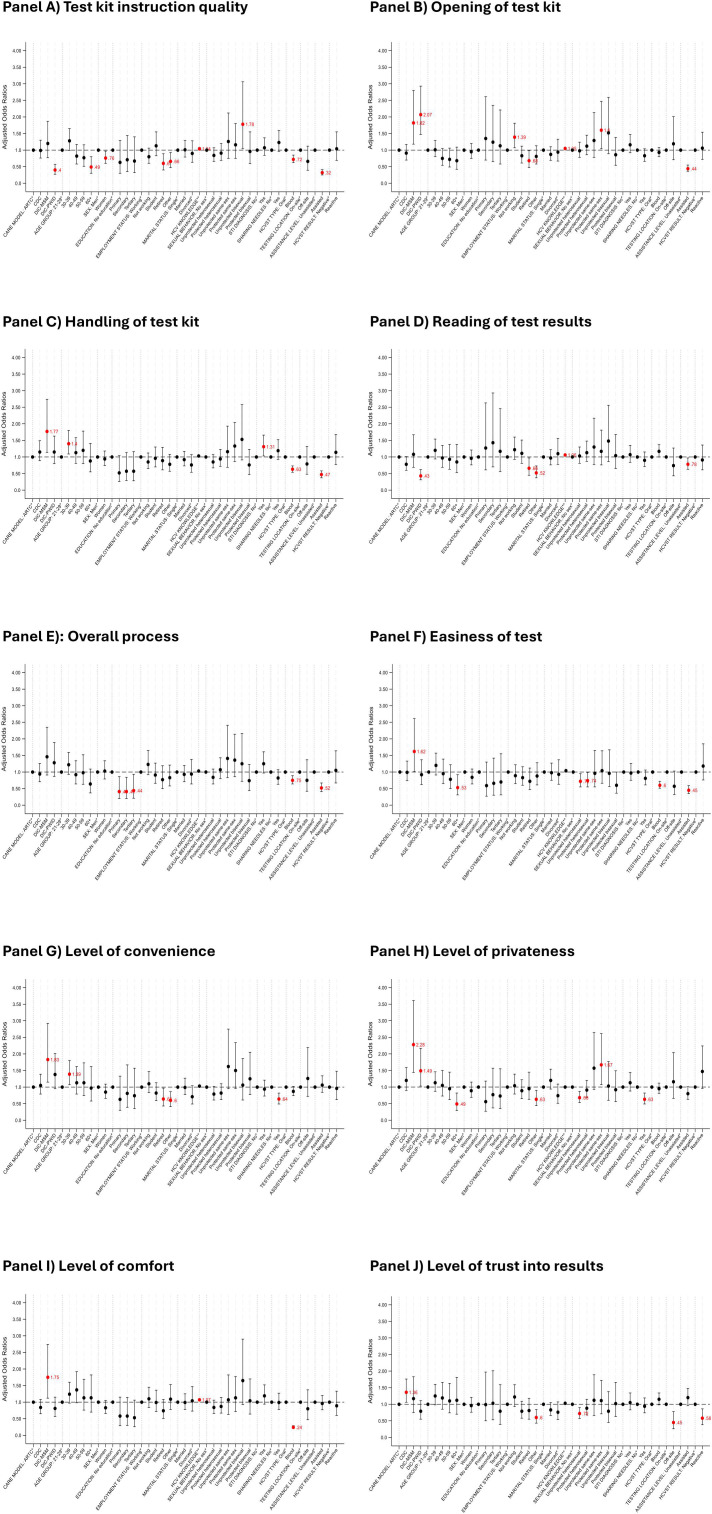
Adjusted odds ratio (aOR) estimates for different aspects of HCV self-testing, derived from ordered logistic regression models. ARTC, antiretroviral therapy clinic; CDC, chronic disease clinic; DIC-MSM, drop-in center for men who have sex with men; DIC-PWID, drop-in center for people who inject drugs. Panels represent: (A) test kit instruction quality, (B) opening of the test kit, (C) handling of the test kit, (D) reading of test results, (E) overall process, (F) easiness of the test, (G) level of convenience, (H) level of privacy, (I) level of comfort, and (J) level of trust in test results. *This is the reference category. **HCV knowledge was modeled as a continues variable.

Several key factors were associated with differences in the HCVST experience across testing domains (S2 Table, Fig 3).

Care model emerged as a strong determinant, with MSM-DIC participants reporting more favourable experiences than those at ARTC, including higher satisfaction with kit opening (adjusted odds ratio [aOR]: 1.82; 95% confidence interval [CI]: 1.18-2.80) and handling (aOR: 1.77; 1.14-2.74). In contrast, PWID-DIC participants had lower odds of understanding test instructions (aOR: 0.40; 0.27-0.57) and reading results (aOR: 0.43; 0.31-0.62), despite reporting good privacy (aOR: 1.49; 1.03-2.17). Assisted HCVST was consistently associated with lower odds of positive ratings across most domains compared to unassisted testing, including ease of use (aOR: 0.45; 0.35-0.59) and satisfaction with the overall testing process (aOR: 0.52; 0.41-0.67). Blood-based HCVST also showed lower odds of favourable responses regarding test instructions, kit handling, the overall testing process, ease, and comfort while it slightly increased odds of successfully reading of test results (aOR: 1.17; 1.00-1.37). Importantly, higher levels of knowledge about HCV were positively associated with improved experiences in 7 out of 10 domains.

Age had a nuanced association. Compared to the 21–29 years group, participants aged 30–39 years tended to report higher satisfaction with kit handling, convenience, and comfort while those aged ≥60 years showed consistently lower satisfaction across testing instructions, overall process, ease, and privacy. Similarly, retired clients had less favourable experiences across 4 out of 10 domains.

Education level showed limited associations across domains with higher education levels linked to less favourable experiences with the overall process and comfort. Off-site testing (aOR: 0.45; 0.26-0.78) and a reactive HCVST result (aOR: 0.58; 0.38-0.86) were only associated with lower trust in test results. Sex showed only minor differences with women presenting with lower odds of easily understanding instructions (aOR: 0.76; 0.60-0.97), and clients who shared needles reporting reduced convenience and privacy. Sexual behaviour patterns contributed nuanced findings, but no consistent trends emerged.

#### Associations with overall satisfaction with HCVST.

Findings from the summary model overall aligned with domain-specific models (Table 4). Participants aged 30–39 years and those with greater HCV knowledge were more likely to report favorable experiences (aOR: 1.34, 1.02-1.77; aOR: 1.07, 1.04-1.11, respectively), whereas retired participants were less likely than employed individuals to do so (aOR: 0.65, 0.44-0.96). Blood-based self-tests (aOR: 0.59, 0.50-0.70) and assisted testing (aOR: 0.32, 0.25-0.40) were both associated with lower satisfaction compared to oral-fluid tests and unassisted testing, respectively.

**Table 4:** Associations with overall satisfaction with HCV self-testing from the generalized ordered logistic regression model assuming proportional odds.

	cOR (95% CI) (n = 2641)^a^	p-value	aOR (95% CI) (n = 2641)^a,b^	p-value
**Care model**				
ARTC	1		1	
CDC	1.07 (0.85-1.36)	0.558	1.21 (0.91-1.60)	0.187
DIC-MSM	3.47 (2.69-4.47)	<0.001	1.98 (1.19-3.27)	0.008
DIC-PWID	1.64 (1.30-2.08)	<0.001	1.73 (1.18-2.53)	0.005
**Age group, years**				
21-29	1		1	
30-39	1.03 (0.80 - 1.32)	0.821	1.34 (1.02-1.77)	0.039
40-49	0.62 (0.48 - 0.79)	<0.001	1.02 (0.70-1.47)	0.936
50-59	0.55 (0.43 - 0.71)	<0.001	1.03 (0.66-1.58)	0.910
≥60	0.31 (0.24 - 0.40)	<0.001	0.82 (0.49-1.37)	0.444
**Sex**				
Men	1		1	
Women	0.50 (0.42 - 0.60)	<0.001	0.85 (0.66-1.10)	0.208
**Education completed**				
No education	1		1	
Primary	0.92 (0.46 - 1.83)	0.812	0.85 (0.41-1.75)	0.651
Secondary	1.88 (0.96 - 3.67)	0.065	1.12 (0.55-2.29)	0.746
Tertiary	2.63 (1.33 - 5.19)	0.005	1.14 (0.54-2.41)	0.727
**Employment status**				
Working	1		1	
Not working	0.63 (0.48 - 0.83)	0.001	0.82 (0.61-1.10)	0.195
Student	1.39 (1.07 - 1.80)	0.013	0.72 (0.51-1.03)	0.075
Retired	0.42 (0.31 - 0.56)	<0.001	0.65 (0.44-0.96)	0.031
Other	0.54 (0.38 - 0.74)	<0.001	0.73 (0.51-1.05)	0.086
**Marital status**				
Single	1		1	
Married	0.59 (0.49 - 0.71)	<0.001	0.82 (0.63-1.06)	0.127
Divorced	0.37 (0.29 - 0.49)	<0.001	0.74 (0.51-1.06)	0.104
**HCV knowledge** ^**c**^	1.07 (1.04 - 1.10)	<0.001	1.07 (1.04-1.11)	<0.001
**Sexual behavior (past 6 months)** ^**d**^				
No sexual contact	1		1	
Unprotected heterosexual contact	1.68 (1.35 - 2.09)	<0.001	1.07 (0.83-1.39)	0.608
Protected heterosexual contact	1.37 (1.08 - 1.75)	0.010	0.89 (0.66-1.19)	0.421
Unprotected same-sex contact	4.47 (2.78 - 7.21)	<0.001	1.49 (0.82-2.71)	0.195
Protected same-sex contact	4.45 (3.11 - 6.38)	<0.001	1.49 (0.89-2.47)	0.128
Unprotected bisexual contact	3.95 (2.43 - 6.43)	<0.001	1.19 (0.64-2.22)	0.582
Protected bisexual contact	2.46 (1.61 - 3.76)	<0.001	0.81 (0.46-1.42)	0.458
**STI diagnosis (past 6 months)**				
No	1		1	
Yes	1.41 (1.09 - 1.82)	0.009	1.09 (0.83-1.42)	0.538
**Sharing needles (past 6 months)**				
No	1		1	
Yes	1.08 (0.88 – 1.33)	0.459	0.93 (0.72-1.21)	0.595
**Type of HCV self-test**				
Oral	1		1	
Blood	0.60 (0.51 - 0.71)	<0.001	0.59 (0.50-0.70)	<0.001
**Location of HCV self-testing**				
On-site	1		1	
Off-site	0.57 (0.34 - 0.95)	0.031	0.72 (0.41-1.26)	0.249
**Level of assistance needed**				
Unassisted	1		1	
Assisted	0.31 (0.25 - 0.38)	<0.001	0.32 (0.25-0.40)	<0.001
**HCVST screening interpretation** ^**e**^				
Negative	1		1	
Reactive	0.62 (0.42 - 0.90)	0.013	1.14 (0.75-1.73)	0.548

aOR, adjusted odds ratio; ARTC, antiretroviral therapy clinic; CI, confidence interval; CDC, chronic disease clinic; cOR, crude odds ratio; DIC-MSM, drop-in center for men who have sex with men; DIC-PWID, drop-in center for people who inject drugs; HCV, hepatitis C virus; HCVST, hepatitis C virus self-testing; STI, sexually transmitted infection.

^a^Three clients were excluded from the multivariable analysis due to missing data on their testing experience.

^b^No variables were excluded due to possible collinearity. Collinearity and confounding were considered during model interpretation by monitoring for wide confidence intervals and non-significant estimates among conceptually related variables, such as care model, type of self-test, level of assistance, and sexual contact type. These factors may share underlying associations (e.g., self-test type and testing assistance), which could influence effect estimates.

^c^Current HCV knowledge was assessed using 8 questions. Participants who had never heard of HCV were assigned a score of 0, while others received cumulative scores based on correct answers. Scores ranged from 0 to 8, with higher scores indicating greater knowledge, and were treated as a continuous variable.

^d^Sexual behavior categories were constructed by combining reported condom use and the gender of sexual partners in the past 6 months. Participants reporting no sexual contact were classified accordingly. Among sexually active individuals, those who reported ‘never’ or ‘rarely’ using condoms were categorized as having unprotected sex, while those reporting condom use ‘often’ or ‘always’ were categorized as having protected sex. This composite variable reflects sexual behaviour but not sexual orientation or gender identity.

^e^Due to the low number of observations in some categories of this variable, the categories “test did not function,” “unable to read result,” and “unwilling to disclose result” were combined with the “non-reactive HCVST result” category. This adjustment was made to improve interpretation and facilitate the calculation of regression outcomes.

**Fig 4:**
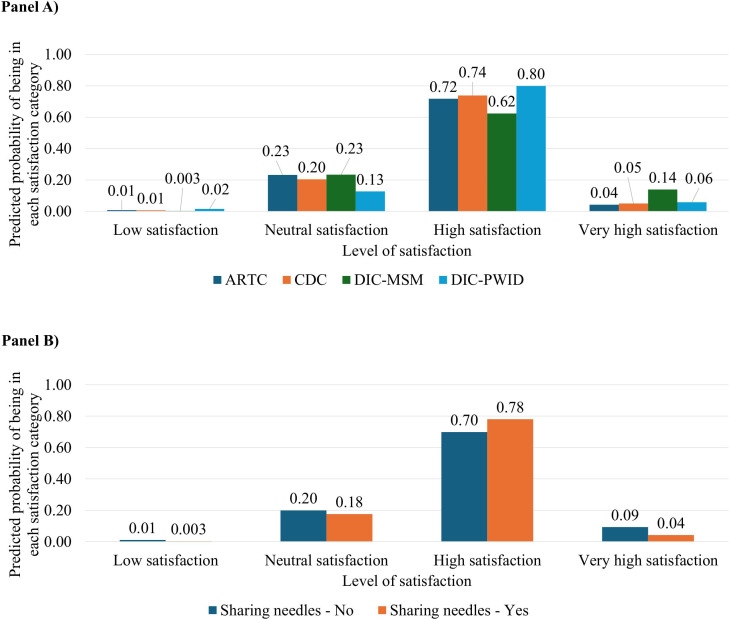
Model‑adjusted predicted satisfaction with HCV self‑testing across care models and needle‑sharing behavior.

Overall, clients at the DIC-MSM (aOR 1.98, 95% CI 1.19-3.27) and DIC-PWID (aOR 1.73, 1.18-2.53) sites were more likely to report favorable experiences than those at ARTC (Table 4). However, the secondary analysis that relaxed the proportional odds assumption showed that these relationships varied by satisfaction level and were largely driven by associations at the top tiers of the satisfaction scale. For instance, compared with ARTC clients, those at DIC-PWID were about twice as likely to report combined high or very high satisfaction levels (aOR 2.04, 1.33-3.14), and those at DIC-MSM were nearly four times as likely to report very high satisfaction (aOR 3.85, 2.21-6.72), while no significant differences were seen between care models at lower satisfaction levels. In addition, while needle sharing was not associated with overall satisfaction (aOR 0.93, 0.72-1.21) in the main model, the secondary analysis demonstrated that needle sharing had 60% lower odds of being in the very high satisfaction group (aOR 0.40, 0.22-0.72) compared to ARTC and with no differences seen at lower levels. These patterns are mirrored and further detailed in the predicted probability bar charts of Fig 4.

ARTC, antiretroviral therapy clinic; CDC, chronic disease clinic; DIC-MSM, drop-in center for men who have sex with men; DIC-PWID, drop-in center for people who inject drugs. Predicted probabilities were obtained from a multivariable generalized ordered logistic regression fitted using the gologit2 command. The model relaxed the proportional‑odds assumption for care model and needle sharing because these predictors showed non‑parallel effects across satisfaction levels. Panel A shows the expected distribution of satisfaction categories for each care model; Panel B contrasts participants who shared needles in the past six months with those who did not. Numbers above the bars indicate the predicted probability of falling into each satisfaction category; within each group the four bars sum to 1. All probabilities are adjusted for the other covariates in the model. In summary, the “high” satisfaction category dominates across care models and needle‑sharing groups, whereas “low” satisfaction is rare. Clients at DIC‑MSM have a markedly larger probability of being in the “very high” category, those at DIC‑PWID have a higher probability of being in the “high” category, and needle sharers have a reduced probability of falling into the “very high” satisfaction category. Differences between groups at the low‑satisfaction level are less informative because of the very small probabilities involved.

#### Perceived advantages of HCV self-testing.

**Fig 5:**
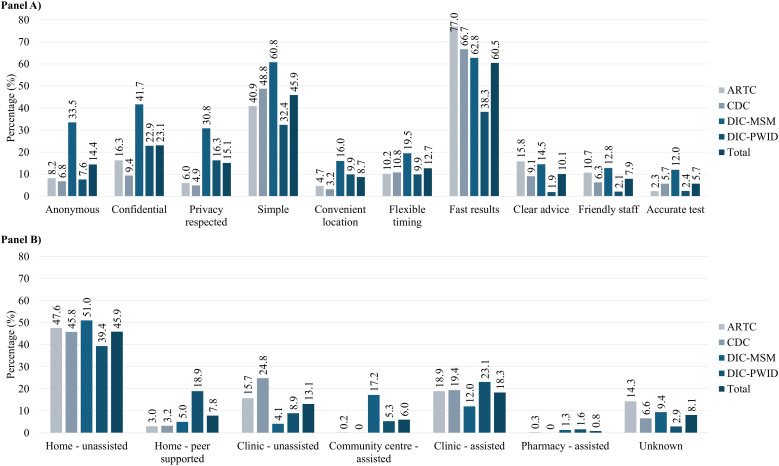
Cited advantages of (Panel A) and preferred future approaches to (Panel B) HCV self-testing by care model and among all clients. For Panel A, clients could select more than one option, so column percentages are not equal to 100%. Each percentage reflects how frequently each category was cited by each client, calculated as a proportion of the total number of participants in each model of care and overall.

Fig 5 (Panel A) and S3 Table summarizes the key perceived advantages of HCVST. The most commonly cited attributes were fast time to result (60.4%), simplicity of use (45.8%), and confidentiality (23.1%), with notable variations across care models. Confidentiality was cited most frequently at the MSM-DIC (41.7%) but less so at clinic-based sites (≤16.3%). In contrast, fast turn-around time was emphasized in clinic settings (e.g., ARTC: 77.0%) but least cited at the PWID-DIC (38.3%). Simplicity was most frequently cited at the MSM-DIC (60.8%) and least at the PWID-DIC (32.4%).

Other perceived advantages, both overall and by care model, were cited by fewer than 20% of participants (Fig 5, Panel A). However, anonymity (33.5%) and respect for privacy (30.8%) were highlighted by a substantial proportion of MSM-DIC clients. The mean number of advantages cited per participant was 2.0, highest at the MSM-DIC (3.0) and lowest at the PWID-DIC (1.4).

#### Recommendations and preferences.

Almost all participants (n = 2606, 98.7%) indicated they would recommend HCV self-testing, with 0.6% (n = 15) neutral and 0.8% (n = 20) not recommending it, distributed across ARTC (n = 8), CDC (n = 3), and PWID-DIC (n = 9) sites (S3 Table).

Fig 5 (Panel B) and S3 Table present preferences for future HCVST use overall and by care model. Unassisted home-based HCV self-testing was the most preferred approach (45.9%) consistently across models (ranging from 39.4% at the PWID-DIC to 51.0% at the MSM-DIC). Other preferences were less common with 18.3% favoring assisted self-testing at a clinic and 13.1% favoring unassisted clinic-based testing; preferences for pharmacy-based assisted testing were negligible (0.8%).

Care model-specific variations included greater preference for peer-supported home testing at the PWID-DIC (18.9% vs. 7.8% overall), unassisted clinic testing at the CDC (24.8% vs. 13.1% overall), and assisted self-testing at the MSM-DIC (17.2% vs. 6.0% overall).

### Feasibility

#### Test interpretation.

Most participants (n = 2562, 96.9%) successfully reported an HCVST result. Difficulties in result interpretation occurred in 82 participants (3.1%), including 2 (0.1%) unwilling to disclose a result, 14 (0.5%) unable to read results, and 66 (2.5%) reporting perceived test failures, with higher proportions of reported failures at ARTC and DIC-MSM sites (p < 0.001) (Table 3).

In multivariable analysis (S4 Table), higher levels of education (e.g., secondary education: aRR: 0.29, 0.09-0.95) vs. no education and use of blood-based HCVST (aRR: 0.31, 0.19-0.52) were associated with lower likelihood of interpretation difficulties while off-site self-testing increased the likelihood (aRR: 3.76, 1.69-8.36).

#### HCV care cascade.

**Fig 6:**
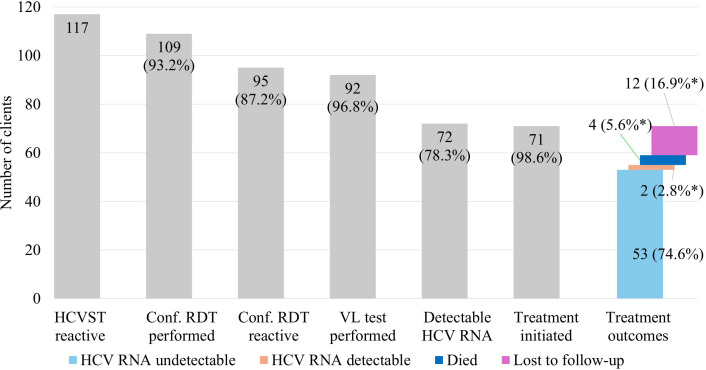
Care cascade for clients with reactive HCV self-testing results (n = 117) through to HCV cure. Conf. RDT, confirmatory rapid diagnostic test for HCV infection; HCVST, hepatitis C virus self-testing; RNA, ribonucleic acid. Percentages along the grey bars are calculated sequentially along the care cascade, using the absolute number from the preceding bar as the denominator. Percentages for treatment outcomes are calculated based on the total number of clients initiated on HCV therapy (n = 71).

Among 117 participants with reactive HCVST results, 93.2% linked to confirmatory rapid diagnostic testing, with 87.2% (n = 95) confirmed antibody-positive (Fig 6). Of these, 92 (96.8%) underwent quantitative HCV RNA testing of whom 78.3% (n = 72) had detectable RNA (current HCV infection) and were considered treatment-eligible, with a median HCV RNA load of 648,451 IU/mL (IQR: 25,519–2,584,451) and a median aspartate aminotransferase to platelet ratio index (APRI) score of 0.4 (IQR: 0.3–0.7). Among these clients, 98.6% (n = 77/78) initiated therapy. Among clients with undetectable HCV RNA, two had APRI scores >0.5, indicating fibrosis. At 3–6 months post-treatment initiation, 74.6% achieved cure (undetectable HCV RNA), two experienced treatment failure, four died, and 12 were lost to follow-up despite tracing efforts. In multivariable analysis (S5 Table), men had higher odds of attrition along the care cascade compared to women (aOR: 3.77, 1.10-12.91). No other clear associations were observed with other baseline factors, including the care model.

### Additional analysis

#### Secondary distribution.

Among 10 index clients with a reactive HCVST result at MSM and PWID drop-in centers, 5 sexual and/or injecting partners were enrolled. All partners performed unassisted blood-based HCVST, either on-site (n = 3) or off-site (n = 2). Participants ranged in age from 21 to 51 years; three were PWID, one was female, and all had at least secondary education. Four were single and employed, with all of them having a minimum of secondary education. HCVST was rated favorably across all domains except for lower intention to recommend. Unassisted, home-based self-testing was the preferred future option (n = 4). Confidentiality, simplicity of procedures, and rapid results were the most frequently cited advantages (n = 3 each).

## Discussion

This study is one of the first to evaluate HCVST delivered through differentiated service models in West and Central Africa, demonstrating high uptake and acceptability among PLHIV, MSM, PWID, and older adults. Findings highlight the feasibility of integrating HCVST into community and healthcare settings, with most participants finding self-testing easy and willing to recommend it. However, limitations in linkage to care and treatment completion emphasize the need to strengthen post-testing pathways as HCVST scales up.

### Feasibility and acceptability of HCV self-testing

High uptake across care models and diverse populations underscores HCVST as an acceptable approach to increasing HCV status awareness. Both oral-fluid and blood-based tests were well accepted, with minor variations by care model and subpopulation. These results align with studies from South Africa and China supporting the usability of self-tests among lay persons, particularly among key populations such as MSM [8,9,15] and reinforce WHO recommendations promoting HCVST alongside provider-administered testing [14]. Although most HCVST occurred on-site, strong preferences for unassisted home-based HCVST suggest high potential for off-site expansion. This is critical for reaching populations outside conventional healthcare settings. However, additional support may be necessary for specific groups such as PWID, who experienced more difficulties during self-testing. Tailored assistance and differentiated delivery models will be key to equitable scale-up. Participants at the clinic sites prioritized rapid turnaround times, while clients at DICs emphasized privacy and confidentiality, likely reflecting concerns about stigma and discrimination. These differences suggest that messaging strategies should be tailored to address subpopulation-specific needs and preferences to optimize uptake.

Secondary distribution of HCVST to partners of clients with confirmed HCV infection was limited, with only five contacts enrolled despite high acceptance among those who tested. Barriers likely included limited eligibility among index cases and stigma surrounding HCV infection. Similar challenges have been reported in index testing for HIV and STIs [27,28], with provider-assisted partner notification recommended to enhance reach and promote disclosure [29,30]. Further research is needed to optimize secondary distribution strategies within HCVST programs.

### Linkages to HCV care

Linkage to confirmatory testing and treatment following HCVST was generally high and acceptable for public health programs. Although based on small numbers, linkage appeared slightly higher from community-based models compared to facility-based sites, despite most HCV-positive cases being identified at facilities. Active follow-up at DICs likely contributed to stronger linkage outcomes, whereas the absence of peer or NGO support and limited integration pathways at facilities may have hindered engagement. Strengthening linkages remains critical to achieving the 2023 HCV elimination targets, necessitating focused health system interventions [31]. Experiences from countries like Rwanda and Georgia show that integrated and decentralized HCV services can significantly improve linkage and support national and global elimination goals [32].

While not a primary focus of this study, men were more likely to experience attrition along the HCV care cascade. Evidence on differences by sex in HCV care or similar interventions such as HIV self-testing remains limited or inconsistent across high and low-income settings, where stigma including gender roles and norms as well structural barriers likely intersect to affect outcomes [33–38]. While further research on these dynamics is warranted, our findings suggest that tailored support for men may be necessary to improve retention and ensure equitable benefits from HCVST in this setting.

### Impact for policy

This study suggests that HCVST can serve as a feasible and critical entry point into the HCV care continuum. To maximize public health impact, policies must strengthen each stage of the pathway. Lessons from HIV programs emphasize the need to expand testing, reduce stigma, and enhance acceptance. Task shifting and task sharing—reallocating simpler tasks to lay health workers—can optimize human resources for health by enabling non-specialists, such as nurses, to initiate DAAs therapy in doctor-scarce settings, while lay cadres expand access to HCVST [39], consequently optimizing healthcare resources. To further expand access and drawing on lessons from HIV self-testing, additional implementation strategies could include mobile testing units for reaching remote or high-risk populations, innovative digital tools (e.g., AI chatbot support) to enhance engagement and usability, and policies enabling community-based distribution and linkage [30,40–42]. Integrating HCVST into existing HIV self-testing, STI, or chronic disease platforms could further optimise reach and serive efficiency.

Policies promoting differentiated care models at both community and facility levels can better reach high-risk populations by offering flexible, person-centered options and reducing structural barriers [30]. Such approaches are essential for advancing national and global HCV elimination goals.

Aligned with the WHO vision for self-care [43], programs should reinforce client autonomy by transitioning from assisted to unassisted HCVST where appropriate, particularly for routine retesting and clients with higher health literacy. Expanding off-site testing, broadening HCVST availability across sectors, and strengthening index testing can further empower individuals, enhance accessibility, and improve program sustainability.

### Limitations

This study focused on specific populations (PLHIV, MSM, PWID, and older adults) accessing differentiated care models, providing valuable operational insights for resource-limited settings. However, the exclusion of other lower-risk groups limits the generalizability to the broader population, which is relevant for national screening efforts. Still, the study identifies where HCVST integration could be most impactful, particularly among elderly populations and key populations.

Findings reflect short-term acceptability and feasibility but do not capture the long-term sustainability of HCVST use. Future research should explore whether high-risk populations, such as PWID, shift towards unassisted testing over time, enhancing self-care models.

The study did not explore barriers and facilitators to linkage to care, a critical component for maximizing HCVST impact. Further research should build on lessons from HIV self-testing programs to strengthen linkage pathways.

Cost-effectiveness analysis was beyond the study’s scope but remains critical for informing policy. Existing evidence suggests cost-effectiveness improves in higher-prevalence populations [19], supporting the feasibility of targeted, differentiated approaches as HCVST kit prices decline.

While the sample size supported multivariable modeling, the use of multiple logit models raises the risk of Type I error. Predictor selection was theory-driven, but findings with marginal significance should be interpreted cautiously.

A key strength of the study was its use of differentiated service delivery models, providing evidence to support scalable, context-adapted HCVST integration across both community and clinic settings.

## Conclusions

This study highlights the feasibility, acceptability, and suggests potential for scalability of HCVST for improving access to HCV care across diverse populations in Cameroon. By integrating HCVST within differentiated community and facility-based care models, we demonstrated its effectiveness in reaching high-risk groups, empowering individuals, and enabling earlier diagnosis and treatment. To fully realize the impact of HCVST, tailored linkage-to-care and support models are critical, especially for populations facing stigma and access barriers. These insights can inform policies to expand HCVST, advancing progress toward the WHO’s 2030 elimination targets in resource-limited settings.

## Supporting information

S1 Table HCV self-testing experience among study participants. (PDF)

S2 Table Domain specific associations with HCV self-testing experience (Panel A) and related qualities (Panel B). (PDF)

S3 Table Perceptions and choices towards HCV self-testing among all clients (n = 2644) and by model of care. (PDF)

S4 Table Univariate and multivariable predictors of difficulties in interpreting HCVST results. (PDF)

S5 Table Univariate and multivariable predictors of cumulative loss to care from reactive HCVST result to HCV cure (n = 117). (PDF)
