# Supplementary material for: Assessing hepatitis C self-testing within differentiated care models in Cameroon: Feasibility, acceptability, and linkage to care for key and priority populations
Source: PLOS Glob Public Health. 2025 Dec 15;5(12):e0005423. doi: 10.1371/journal.pgph.0005423 (PMC12704849; doi:10.1371/journal.pgph.0005423)
Supplement: S1 Table — (PDF) [file pgph.0005423.s001.pdf]

S1 Table. HCV self-testing experience among study participants.

	Total	ARTC	CDC	DIC-MSM	DIC-PWID	p
**Test instructions**						
Very difficult	12 (0.5)	1 (0.2)	1 (0.2)	5 (0.7)	5 (0.7)	<0.001
Difficult	133 (5.0)	25 (4.2)	31 (4.8)	9 (1.3)	68 (9.7)	
Neutral	121 (4.6)	41 (6.9)	30 (4.6)	20 (2.8)	30 (4.3)	
Easy	1645 (62.3)	370 (62.4)	468 (72.2)	320 (45.6)	487 (69.8)	
Very easy	730 (27.6)	156 (26.3)	118 (18.2)	348 (49.6)	108 (15.5)	
**Opening of test kit**						
Very difficult	10 (0.4)	3 (0.5)	3 (0.5)	0 (0.0)	4 (0.6)	<0.001
Difficult	255 (9.7)	91 (15.3)	108 (16.7)	41 (5.8)	15 (2.1)	
Neutral	122 (4.6)	20 (3.4)	26 (4.0)	29 (4.1)	47 (6.7)	
Easy	1483 (56.2)	352 (59.4)	422 (65.1)	312 (44.4)	397 (56.9)	
Very easy	771 (29.2)	127 (21.4)	89 (13.7)	320 (45.6)	235 (33.7)	
**Handling of test kit**						
Very difficult	16 (0.6)	3 (0.5)	2 (0.3)	7 (1.0)	4 (0.6)	<0.001
Difficult	160 (6.1)	34 (5.7)	41 (6.3)	18 (2.6)	67 (9.6)	
Neutral	198 (7.5)	62 (10.5)	47 (7.3)	54 (7.7)	35 (5.0)	
Easy	1602 (60.7)	388 (65.4)	449 (69.3)	339 (48.3)	426 (61.0)	
Very easy	665 (25.2)	106 (17.9)	109 (16.8)	284 (40.5)	166 (23.8)	
**Reading of results**						
Very difficult	5 (0.2)	1 (0.2)	1 (0.2)	2 (0.3)	1 (0.1)	<0.001
Difficult	64 (2.4)	8 (1.3)	21 (3.2)	15 (2.1)	20 (2.9)	
Neutral	64 (2.4)	26 (4.4)	12 (1.9)	15 (2.1)	11 (1.6)	
Easy	1423 (53.9)	279 (47.0)	383 (59.1)	274 (39.0)	487 (69.8)	
Very easy	1085 (41.1)	279 (47.0)	231 (35.6)	396 (56.4)	179 (25.6)	
**Overall testing process**						
Very difficult	3 (0.1)	1 (0.2)	0 (0.0)	0 (0.0)	2 (0.3)	<0.001
Difficult	58 (2.2)	11 (1.9)	16 (2.5)	7 (1.0)	24 (3.4)	
Neutral	90 (3.4)	22 (3.7)	23 (3.5)	23 (3.3)	22 (3.2)	
Easy	1868 (70.7)	444 (74.9)	519 (80.1)	424 (60.4)	481 (68.9)	
Very easy	622 (23.6)	115 (19.4)	90 (13.9)	248 (35.3)	169 (24.2)	
**Level of easiness**						
Very low	4 (0.2)	1 (0.2)	0 (0.0)	1 (0.1)	2 (0.3)	<0.001
Low	72 (2.7)	13 (2.2)	19 (2.9)	6 (0.9)	34 (4.9)	
Neutral	96 (3.6)	23 (3.9)	21 (3.2)	37 (5.3)	15 (2.1)	
High	1910 (72.3)	457 (77.1)	524 (80.9)	395 (56.3)	534 (76.5)	
Very high	559 (21.2)	99 (16.7)	84 (13.0)	263 (37.5)	113 (16.2)	
**Level of convenience**						
Very low	1 (0.0)	0 (0.0)	0 (0.0)	0 (0.0)	1 (0.1)	<0.001
Low	17 (0.6)	3 (0.5)	1 (0.2)	1 (0.1)	12 (1.7)	
Neutral	167 (6.3)	52 (8.8)	37 (5.7)	51 (7.3)	27 (3.9)	
High	1784 (67.6)	424 (71.5)	515 (79.5)	351 (50.0)	494 (70.8)	
Very high	672 (25.4)	114 (19.2)	95 (14.7)	299 (42.6)	164 (23.5)	
**Level of privacy**						
Very low	2 (0.1)	1 (0.2)	0 (0.0)	1 (0.1)	0 (0.0)	<0.001
Low	9 (0.3)	2 (0.3)	4 (0.6)	1 (0.1)	2 (0.3)	
Neutral	109 (4.1)	37 (6.2)	35 (5.4)	27 (3.8)	10 (1.4)	

High	1690 (64.0)	424 (71.5)	465 (71.8)	301 (42.9)	500 (71.6)	
Very high	831 (31.5)	129 (21.8)	144 (22.2)	372 (53.0)	186 (26.6)	
**Comfort with process**						
Very low	8 (0.3)	1 (0.2)	1 (0.2)	3 (0.4)	3 (0.4)	<0.001
Low	111 (4.2)	18 (3.0)	47 (7.3)	15 (2.1)	31 (4.4)	
Neutral	125 (4.7)	45 (7.6)	21 (3.2)	48 (6.8)	11 (1.6)	
High	1466 (55.5)	320 (54.0)	403 (62.2)	266 (37.9)	477 (68.3)	
Very high	931 (35.3)	209 (35.2)	176 (27.2)	370 (52.7)	176 (25.2)	
**Trust in results**						
Very low	7 (0.3)	6 (1.0)	1 (0.2)	0 (0.0)	0 (0.0)	<0.001
Low	11 (0.4)	2 (0.3)	3 (0.5)	5 (0.7)	1 (0.1)	
Neutral	168 (6.4)	27 (4.6)	27 (4.2)	97 (13.8)	17 (2.4)	
High	1554 (58.8)	373 (62.9)	390 (60.2)	295 (42.0)	496 (71.1)	
Very high	901 (34.1)	185 (31.2)	227 (35.0)	305 (43.4)	184 (26.4)	

ARTC, antiretroviral therapy clinic; CDC, chronic disease clinic; CI, confidence interval; DIC-MSM, drop-in center for men who have sex with men; DIC-PWID, drop-in center for people who inject drugs; p, p-value.
