# Supplementary material for: Assessing hepatitis C self-testing within differentiated care models in Cameroon: Feasibility, acceptability, and linkage to care for key and priority populations
Source: PLOS Glob Public Health. 2025 Dec 15;5(12):e0005423. doi: 10.1371/journal.pgph.0005423 (PMC12704849; doi:10.1371/journal.pgph.0005423)
Supplement: S2 Table — (PDF) [file pgph.0005423.s002.pdf]

S2 Table. Domain specific associations with HCV self-testing experience (Panel A) and related qualities (Panel B).

Panel A)

	Test instructions		Opening of test kit		Handling of test kit		Reading of results		Overall testing process	
	**aOR (95% CI)**	**p**	**aOR (95% CI)**	**p**	**aOR (95% CI)**	**p**	**aOR (95% CI)**	**p**	**aOR (95% CI)**	**p**
**Care model**										
ARTC	1		1		1		1		1	
CDC	0.99 (0.76 - 1.3)	0.953	0.91 (0.7 - 1.17)	0.455	1.15 (0.89 - 1.49)	0.271	0.78 (0.6 - 1.01)	0.057	0.94 (0.71 - 1.26)	0.698
DIC-MSM	1.20 (0.77 - 1.87)	0.432	1.82 (1.18 - 2.80)	0.007	1.77 (1.14 - 2.74)	0.011	1.08 (0.69 - 1.67)	0.736	1.46 (0.91 - 2.35)	0.114
DIC-PWID	0.40 (0.27 - 0.57)	<0.001	2.07 (1.47 - 2.93)	<0.001	1.15 (0.8 - 1.63)	0.451	0.43 (0.31 - 0.62)	<0.001	1.28 (0.87 - 1.89)	0.211
**Age group, years**										
21-29	1		1		1		1		1	
30-39	1.28 (0.99 - 1.65)	0.061	1.01 (0.8 - 1.29)	0.919	1.4 (1.09 - 1.8)	0.008	1.2 (0.94 - 1.54)	0.149	1.22 (0.93 - 1.59)	0.150
40-49	0.82 (0.58 - 1.17)	0.276	0.75 (0.54 - 1.05)	0.092	1.13 (0.81 - 1.58)	0.473	0.97 (0.69 - 1.35)	0.842	0.92 (0.64 - 1.34)	0.667
50-59	0.77 (0.51 - 1.16)	0.203	0.72 (0.49 - 1.06)	0.100	1.20 (0.80 - 1.78)	0.374	0.93 (0.63 - 1.38)	0.715	0.98 (0.63 - 1.52)	0.939
≥60	0.49 (0.3 - 0.80)	0.005	0.68 (0.42 - 1.09)	0.111	0.88 (0.55 - 1.41)	0.591	0.85 (0.53 - 1.38)	0.517	0.64 (0.37 - 1.09)	0.099
**Sex**										
Men	1		1		1		1		1	
Women	0.76 (0.6 - 0.97)	0.030	0.95 (0.75 - 1.2)	0.659	0.94 (0.74 - 1.18)	0.578	0.96 (0.76 - 1.21)	0.704	1.03 (0.79 - 1.34)	0.821
**Education completed**										
No education	1		1		1		1		1	
Primary	0.63 (0.3 - 1.29)	0.206	1.35 (0.7 - 2.61)	0.367	0.52 (0.26 - 1.04)	0.064	1.27 (0.61 - 2.63)	0.526	0.41 (0.2 - 0.86)	0.017
Secondary	0.71 (0.35 - 1.44)	0.338	1.24 (0.65 - 2.35)	0.518	0.57 (0.29 - 1.13)	0.109	1.43 (0.7 - 2.93)	0.325	0.41 (0.2 - 0.84)	0.015
Tertiary	0.67 (0.32 - 1.4)	0.284	1.13 (0.58 - 2.21)	0.718	0.57 (0.28 - 1.16)	0.120	1.17 (0.56 - 2.46)	0.677	0.44 (0.21 - 0.93)	0.031
**Employment status**										
Working	1		1		1		1		1	
Not working	0.8 (0.6 - 1.06)	0.122	1.39 (1.07 - 1.81)	0.014	0.85 (0.65 - 1.12)	0.254	1.22 (0.93 - 1.6)	0.146	1.23 (0.92 - 1.65)	0.166
Student	1.13 (0.83 - 1.55)	0.437	0.83 (0.61 - 1.12)	0.225	0.96 (0.7 - 1.3)	0.774	1.11 (0.81 - 1.51)	0.520	0.91 (0.66 - 1.27)	0.589
Retired	0.60 (0.4 - 0.89)	0.010	0.68 (0.47 - 0.99)	0.046	0.89 (0.61 - 1.29)	0.540	0.66 (0.45 - 0.97)	0.035	0.77 (0.5 - 1.19)	0.240
Other	0.66 (0.47 - 0.93)	0.017	0.81 (0.58 - 1.13)	0.209	0.78 (0.57 - 1.08)	0.134	0.52 (0.37 - 0.73)	<0.001	0.83 (0.58 - 1.21)	0.338
**Marital status**										
Single	1		1		1		1		1	
Married	1.01 (0.79 - 1.29)	0.943	0.87 (0.69 - 1.1)	0.251	0.92 (0.73 - 1.17)	0.506	0.97 (0.76 - 1.22)	0.776	0.93 (0.71 - 1.2)	0.558
Divorced	0.9 (0.63 - 1.28)	0.549	0.94 (0.67 - 1.33)	0.738	0.76 (0.54 - 1.07)	0.121	1.1 (0.78 - 1.56)	0.591	0.94 (0.64 - 1.38)	0.754
**HCV knowledge[a]**	1.04 (1.01 - 1.08)	0.005	1.05 (1.02 - 1.08)	<0.001	1.03 (1 - 1.06)	0.045	1.06 (1.03 - 1.09)	<0.001	1.03 (1 - 1.06)	0.091
**Sexual behavior (past 6 months)[b]**										

No sexual contact	**1**		1		1		1		1	
Unprotected heterosexual contact	0.84 (0.66 - 1.07)	0.160	0.98 (0.78 - 1.24)	0.890	0.85 (0.67 - 1.07)	0.166	1.03 (0.81 - 1.3)	0.803	0.84 (0.64 - 1.09)	0.181
Protected heterosexual contact	0.91 (0.69 - 1.2)	0.511	1.12 (0.86 - 1.45)	0.411	0.94 (0.72 - 1.23)	0.673	1.13 (0.87 - 1.48)	0.356	1.07 (0.8 - 1.43)	0.668
Unprotected same-sex contact	1.26 (0.75 - 2.12)	0.379	1.29 (0.78 - 2.13)	0.324	1.16 (0.69 - 1.93)	0.575	1.3 (0.77 - 2.17)	0.323	1.41 (0.83 - 2.41)	0.201
Protected same-sex contact	1.16 (0.75 - 1.80)	0.496	1.60 (1.04 - 2.47)	0.032	1.33 (0.86 - 2.04)	0.195	1.17 (0.76 - 1.81)	0.485	1.36 (0.86 - 2.14)	0.184
Unprotected bisexual contact	1.78 (1.04 - 3.06)	0.036	1.52 (0.89 - 2.59)	0.126	1.53 (0.91 - 2.58)	0.112	1.48 (0.86 - 2.56)	0.153	1.25 (0.72 - 2.16)	0.425
Protected bisexual contact	0.96 (0.59 - 1.55)	0.862	0.86 (0.54 - 1.38)	0.539	0.76 (0.47 - 1.23)	0.262	1.04 (0.65 - 1.68)	0.861	0.74 (0.44 - 1.23)	0.241
**STI diagnosis (past 6 months)**										
No	1		1		1		1		1	
Yes	1.07 (0.84 - 1.37)	0.577	1.16 (0.92 - 1.47)	0.214	1.31 (1.03 - 1.66)	0.030	1.07 (0.84 - 1.35)	0.583	1.25 (0.97 - 1.61)	0.089
**Sharing needles (past 6 months)**										
No	1		1		1		1		1	
Yes	1.23 (0.96 - 1.59)	0.105	0.83 (0.66 - 1.04)	0.110	1.19 (0.93 - 1.52)	0.166	0.9 (0.71 - 1.15)	0.413	0.82 (0.63 - 1.07)	0.140
**Type of HCV self-test**										
Oral	1		1		1		1		1	
Blood	0.72 (0.62 - 0.85)	<0.001	0.93 (0.8 - 1.08)	0.343	0.63 (0.54 - 0.73)	<0.001	1.17 (1.00 - 1.37)	0.047	0.75 (0.64 - 0.89)	0.001
**Location of HCV self-testing**										
On-site	1		1		1		1		1	
Off-site	0.66 (0.39 - 1.12)	0.125	1.19 (0.71 - 2.01)	0.507	0.79 (0.48 - 1.32)	0.373	0.74 (0.43 - 1.27)	0.272	0.75 (0.42 - 1.37)	0.353
**Level of assistance needed**										
Unassisted	1		1		1		1		1	
Assisted	0.32 (0.25 - 0.41)	<0.001	0.44 (0.36 - 0.55)	<0.001	0.47 (0.37 - 0.58)	<0.001	0.78 (0.63 - 0.97)	0.028	0.52 (0.41 - 0.67)	<0.001
**HCVST screening interpretation**[c]										
Negative	1		1		1		1		1	
Reactive	1.04 (0.69 - 1.55)	0.867	1.06 (0.72 - 1.54)	0.775	1.14 (0.77 - 1.68)	0.526	0.91 (0.61 - 1.36)	0.660	1.05 (0.67 - 1.64)	0.832

Panel B)

	**Level of easiness**		**Level of convenience**		**Level of privacy**		**Comfort with process**		**Trust in results**	
	**aOR (95% CI)**	**p**	**aOR (95% CI)**	**p**	**aOR (95% CI)**	**p**	**aOR (95% CI)**	**p**	**aOR (95% CI)**	**p**
**Care model**										
ARTC	1		1		1		1		1	
CDC	0.99 (0.74 - 1.33)	0.961	1.05 (0.79 - 1.39)	0.727	1.2 (0.9 - 1.59)	0.212	0.84 (0.65 - 1.08)	0.178	1.36 (1.06 - 1.76)	0.017
DIC-MSM	1.62 (1.01 - 2.61)	0.047	1.83 (1.15 - 2.92)	0.011	2.28 (1.44 - 3.61)	<0.001	1.75 (1.12 - 2.74)	0.015	1.17 (0.75 - 1.83)	0.487
DIC-PWID	0.92 (0.62 - 1.37)	0.670	1.38 (0.95 - 2.01)	0.090	1.49 (1.03 - 2.17)	0.035	0.81 (0.57 - 1.15)	0.239	0.79 (0.55 - 1.11)	0.174
**Age group, years**										

21-29	1		1		1		1		1	
30-39	1.20 (0.91 - 1.57)	0.200	1.39 (1.07 - 1.8)	0.012	1.13 (0.87 - 1.46)	0.359	1.24 (0.97 - 1.6)	0.09	1.25 (0.98 - 1.6)	0.075
40-49	0.95 (0.65 - 1.39)	0.791	1.13 (0.79 - 1.62)	0.499	1.05 (0.73 - 1.5)	0.800	1.37 (0.98 - 1.92)	0.069	1.19 (0.85 - 1.66)	0.312
50-59	0.78 (0.5 - 1.22)	0.276	1.13 (0.74 - 1.73)	0.564	0.95 (0.62 - 1.45)	0.815	1.13 (0.76 - 1.69)	0.556	1.1 (0.74 - 1.63)	0.635
≥60	0.53 (0.31 - 0.92)	0.024	0.97 (0.58 - 1.62)	0.905	0.49 (0.29 - 0.82)	0.007	1.13 (0.7 - 1.82)	0.621	1.12 (0.7 - 1.81)	0.627
**Sex**										
Men	1		1		1		1		1	
Women	0.84 (0.64 - 1.09)	0.190	0.85 (0.66 - 1.09)	0.197	0.89 (0.69 - 1.15)	0.366	0.83 (0.66 - 1.05)	0.121	0.96 (0.76 - 1.21)	0.737
**Education completed**										
No education	1		1		1		1		1	
Primary	0.59 (0.27 - 1.3)	0.188	0.63 (0.3 - 1.33)	0.224	0.56 (0.27 - 1.18)	0.130	0.58 (0.29 - 1.15)	0.117	0.99 (0.5 - 1.97)	0.985
Secondary	0.66 (0.3 - 1.42)	0.286	0.81 (0.39 - 1.67)	0.565	0.77 (0.37 - 1.57)	0.471	0.58 (0.3 - 1.14)	0.115	1.03 (0.53 - 2.01)	0.938
Tertiary	0.70 (0.31 - 1.55)	0.378	0.74 (0.35 - 1.57)	0.432	0.73 (0.35 - 1.55)	0.419	0.53 (0.26 - 1.06)	0.073	0.79 (0.39 - 1.59)	0.506
**Employment status**										
Working	1		1		1		1		1	
Not working	0.89 (0.65 - 1.22)	0.475	1.1 (0.83 - 1.47)	0.499	1.04 (0.79 - 1.39)	0.768	1.1 (0.84 - 1.45)	0.484	1.22 (0.93 - 1.59)	0.151
Student	0.83 (0.6 - 1.16)	0.269	0.82 (0.59 - 1.13)	0.231	0.89 (0.65 - 1.22)	0.469	0.99 (0.72 - 1.36)	0.947	0.79 (0.58 - 1.09)	0.147
Retired	0.72 (0.47 - 1.12)	0.148	0.64 (0.43 - 0.97)	0.037	0.95 (0.63 - 1.45)	0.828	0.74 (0.51 - 1.08)	0.118	0.81 (0.55 - 1.18)	0.267
Other	0.88 (0.61 - 1.28)	0.518	0.6 (0.41 - 0.86)	0.005	0.63 (0.44 - 0.9)	0.013	1.09 (0.78 - 1.53)	0.596	0.6 (0.43 - 0.84)	0.003
**Marital status**										
Single	1		1		1		1		1	
Married	0.98 (0.75 - 1.27)	0.864	0.99 (0.77 - 1.27)	0.929	1.2 (0.94 - 1.54)	0.145	0.99 (0.78 - 1.25)	0.920	0.83 (0.66 - 1.05)	0.113
Divorced	0.93 (0.63 - 1.37)	0.700	0.71 (0.49 - 1.04)	0.080	0.74 (0.51 - 1.09)	0.131	1.04 (0.73 - 1.47)	0.827	0.76 (0.54 - 1.07)	0.115
**HCV knowledge**[a]	1.04 (1.00 - 1.07)	0.034	1.03 (1.00 - 1.06)	0.063	1 (0.97 - 1.03)	0.900	1.07 (1.04 - 1.10)	<0.001	1.03 (1.00 - 1.06)	0.032
**Sexual behavior (past 6 months)**[b]										
No sexual contact	1		1		1		1		1	
Unprotected heterosexual contact	0.72 (0.55 - 0.95)	0.018	0.79 (0.61 - 1.02)	0.066	0.68 (0.53 - 0.87)	0.003	0.84 (0.66 - 1.06)	0.146	0.72 (0.57 - 0.90)	0.005
Protected heterosexual contact	0.74 (0.55 - 0.99)	0.046	0.82 (0.62 - 1.10)	0.184	0.91 (0.69 - 1.20)	0.510	0.87 (0.67 - 1.14)	0.308	0.88 (0.68 - 1.15)	0.358
Unprotected same-sex contact	0.96 (0.55 - 1.65)	0.872	1.62 (0.95 - 2.75)	0.076	1.57 (0.93 - 2.64)	0.092	1.07 (0.63 - 1.82)	0.795	1.12 (0.66 - 1.89)	0.683
Protected same-sex contact	1.04 (0.66 - 1.64)	0.879	1.5 (0.96 - 2.34)	0.078	1.67 (1.07 - 2.61)	0.023	1.13 (0.72 - 1.77)	0.593	1.11 (0.71 - 1.72)	0.650
Unprotected bisexual contact	0.96 (0.56 - 1.67)	0.897	1.06 (0.61 - 1.86)	0.833	1.03 (0.60 - 1.77)	0.905	1.65 (0.93 - 2.90)	0.085	0.79 (0.45 - 1.38)	0.410
Protected bisexual contact	0.60 (0.36 – 1.00)	0.049	1.25 (0.77 - 2.05)	0.371	0.92 (0.56 - 1.49)	0.728	1.04 (0.64 - 1.70)	0.875	1.02 (0.63 - 1.66)	0.934
**STI diagnosis (past 6 months)**										
No	1		1		1		1		1	
Yes	0.97 (0.75 - 1.26)	0.840	0.94 (0.73 - 1.21)	0.654	1.13 (0.88 - 1.44)	0.342	1.19 (0.94 - 1.52)	0.151	1.05 (0.83 - 1.34)	0.664

**Sharing needles (past 6 months)**										
No	1		1		1		1		1	
Yes	0.81 (0.61 - 1.06)	0.129	0.64 (0.49 - 0.82)	0.001	0.63 (0.49 - 0.82)	<0.001	0.99 (0.78 - 1.27)	0.967	0.94 (0.74 - 1.19)	0.614
**Type of HCV self-test**										
Oral	1		1		1		1		1	
Blood	0.60 (0.51 - 0.72)	<0.001	0.87 (0.74 - 1.02)	0.092	0.95 (0.81 - 1.12)	0.568	0.24 (0.21 - 0.29)	<0.001	1.15 (0.98 - 1.34)	0.083
**Location of HCV self-testing**										
On-site	1		1		1		1		1	
Off-site	0.57 (0.32 - 1.03)	0.062	1.26 (0.72 - 2.19)	0.424	1.16 (0.66 - 2.04)	0.610	0.80 (0.47 - 1.37)	0.420	0.45 (0.26 - 0.78)	0.005
**Level of assistance needed**										
Unassisted	1		1		1		1		1	
Assisted	0.45 (0.35 - 0.59)	<0.001	1.06 (0.84 - 1.34)	0.639	0.8 (0.63 - 1.01)	0.058	0.96 (0.77 - 1.2)	0.725	1.2 (0.96 - 1.48)	0.103
**HCVST screening interpretation**[c]										
Negative	1		1		1		1		1	
Reactive	1.18 (0.76 - 1.85)	0.457	0.96 (0.62 - 1.48)	0.852	1.47 (0.96 - 2.24)	0.076	0.89 (0.6 - 1.33)	0.580	0.58 (0.38 - 0.86)	0.008

aOR, adjusted odds ratio; ARTC, antiretroviral therapy clinic; CDC, chronic disease clinic; CI, confidence interval; DIC-MSM, drop-in center for men who have sex with men; DIC-PWID, drop-in center for people who inject drugs; HCV, hepatitis C virus; HCVST, hepatitis C virus self-testing; STI, sexually transmitted infection; p, p-value.

Three clients were excluded from the multivariable analysis due to missing data on their testing experience. No variables were excluded due to possible collinearity. Collinearity and confounding were considered during model interpretation by monitoring for wide confidence intervals and non-significant estimates among conceptually related variables, such as care model, type of self-test, level of assistance, and sexual contact type. These factors may share underlying associations (e.g. self-test type and testing assistance), which could influence effect estimates.

[a]Current HCV knowledge was assessed using 8 questions. Participants who had never heard of HCV were assigned a score of 0, while others received cumulative scores based on correct answers. Scores ranged from 0 to 8, with higher scores indicating greater knowledge, and were treated as a continuous variable.

[b]Sexual behavior categories were constructed by combining reported condom use and the gender of sexual partners in the past 6 months. Participants reporting no sexual contact were classified accordingly. Among sexually active individuals, those who reported 'never' or 'rarely' using condoms were categorized as having unprotected sex, while those reporting condom use 'often' or 'always' were categorized as having protected sex. This composite variable reflects sexual behaviour but not sexual orientation or gender identity.

[c]Due to the low number of observations in some categories of this variable, the categories "test did not function," "unable to read result," and "unwilling to disclose result" were combined with the "non-reactive HCVST result" category. This adjustment was made to improve interpretation and facilitate the calculation of regression outcomes.
