# Supplementary material for: Assessing hepatitis C self-testing within differentiated care models in Cameroon: Feasibility, acceptability, and linkage to care for key and priority populations
Source: PLOS Glob Public Health. 2025 Dec 15;5(12):e0005423. doi: 10.1371/journal.pgph.0005423 (PMC12704849; doi:10.1371/journal.pgph.0005423)
Supplement: S3 Table — (PDF) [file pgph.0005423.s003.pdf]

S3 Table. Perceptions and choices towards HCV self-testing among all clients (n=2644) and by model of care.

	ARTC	CDC	MSM-DIC	PWID-DIC	All clients
**Recommendation of self-test to others**					
Yes	577 (97.3)	643 (99.2)	700 (99.7)	686 (98.3)	2606 (98.7)
No	8 (1.3)	2 (0.3)	2 (0.3)	3 (0.4)	15 (0.6)
Neutral	8 (1.3)	3 (0.5)	0 (0)	9 (1.3)	20 (0.8)
**Preferred approach to self-testing in future**					
Home & unassisted	282 (47.6)	297 (45.8)	358 (51)	275 (39.4)	1212 (45.9)
Home & peer supported	18 (3)	21 (3.2)	35 (5)	132 (18.9)	206 (7.8)
Unassisted at the clinic	93 (15.7)	161 (24.8)	29 (4.1)	62 (8.9)	345 (13.1)
Assisted at a community centre	1 (0.2)	0 (0)	121 (17.2)	37 (5.3)	159 (6)
Assisted at the clinic	112 (18.9)	126 (19.4)	84 (12)	161 (23.1)	483 (18.3)
Assisted at the pharmacy	2 (0.3)	0 (0)	9 (1.3)	11 (1.6)	22 (0.8)
Undetermined	85 (14.3)	43 (6.6)	66 (9.4)	20 (2.9)	214 (8.1)
**Cited advantages of self-testing[a]**					
Anonymous	49 (8.2)	44 (6.8)	235 (33.5)	53 (7.6)	381 (14.4)
Confidential	97 (16.3)	61 (9.4)	293 (41.7)	160 (22.9)	611 (23.1)
Privacy respected	36 (6)	32 (4.9)	216 (30.8)	114 (16.3)	398 (15.1)
Simple	244 (40.9)	316 (48.8)	427 (60.8)	226 (32.4)	1213 (45.9)
Convenient location	28 (4.7)	21 (3.2)	112 (16)	69 (9.9)	230 (8.7)
Flexible timing	61 (10.2)	70 (10.8)	137 (19.5)	69 (9.9)	337 (12.7)
Fast results	459 (77)	432 (66.7)	441 (62.8)	267 (38.3)	1599 (60.5)
Clear advice	94 (15.8)	59 (9.1)	102 (14.5)	13 (1.9)	268 (10.1)
Friendly staff	64 (10.7)	41 (6.3)	90 (12.8)	15 (2.1)	210 (7.9)
Accurate test	14 (2.3)	37 (5.7)	84 (12)	17 (2.4)	152 (5.7)
**Total number of advantages cited**	1146	1113	2137	1003	5399
**Average number of advantages cited per client[b]**	1.9	1.7	3.0	1.4	2.0

ARTC, antiretroviral therapy clinic; CDC, chronic disease clinic; DIC-MSM, drop-in center for men who have sex with men; DIC-PWID, drop-in center for people who inject drugs; HCVST, hepatitis C virus self-testing.

[a] *Clients were allowed to select more than one option, so column percentages are not equal to 100%. Each percentage reflects how frequently each category was cited by each client, calculated as a proportion of the total number of participants in each model of care and overall.*

[b] *The average number was calculated by dividing the total number of advantages cited—both overall and by model of care—by the number of clients in each model and overall.*
