# Supplementary material for: Assessing hepatitis C self-testing within differentiated care models in Cameroon: Feasibility, acceptability, and linkage to care for key and priority populations
Source: PLOS Glob Public Health. 2025 Dec 15;5(12):e0005423. doi: 10.1371/journal.pgph.0005423 (PMC12704849; doi:10.1371/journal.pgph.0005423)
Supplement: S4 Table — (PDF) [file pgph.0005423.s004.pdf]

S4 Table. Univariate and multivariable predictors of difficulties in interpreting HCVST results.

	cRR (n=2644[2])	p-value	aRR (n=2641[2])	p-value
**Care model**				
ARTC	1		1	
CDC	0.69 (0.38 - 1.26)	0.226	0.64 (0.32 - 1.25)	0.190
DIC-MSM	0.96 (0.56 - 1.64)	0.867	1.81 (0.59 - 5.49)	0.298
DIC-PWID	0.46 (0.24 - 0.90)	0.023	0.98 (0.35 - 2.77)	0.976
**Age group, years**				
21-29	1		1	
30-39	1.00 (0.52 - 1.91)	0.994	1.14 (0.55 - 2.37)	0.732
40-49	1.29 (0.70 - 2.39)	0.415	1.92 (0.76 - 4.86)	0.170
50-59	1.09 (0.56 - 2.11)	0.808	1.77 (0.58 - 5.40)	0.313
≥60	1.36 (0.73 – 2.56)	0.335	2.51 (0.70 - 9.03)	0.159
**Sex**				
Men	1		1	
Women	0.95 (0.60 - 1.51)	0.838	0.82 (0.42 - 1.60)	0.570
**Education completed**				
No education	1		1	
Primary	0.34 (0.10 - 1.18)	0.090	0.32 (0.09 - 1.11)	0.072
Secondary	0.34 (0.11 - 1.04)	0.058	0.29 (0.09 - 0.95)	0.041
Tertiary	0.45 (0.14 - 1.42)	0.173	0.28 (0.08 - 1.02)	0.053
**Employment status**				
Working	1		1	
Not working	0.56 (0.23 - 1.40)	0.215	0.67 (0.26 - 1.69)	0.392
Student	1.19 (0.67 - 2.12)	0.556	1.22 (0.55 - 2.72)	0.627
Retired	1.21 (0.58 - 2.50)	0.616	1.02 (0.40 - 2.65)	0.960
Other	0.55 (0.17 - 1.73)	0.305	0.45 (0.14 - 1.45)	0.178
**Marital status**				
Single	1		1	
Married	1.28 (0.80 - 2.04)	0.310	1.16 (0.59 - 2.28)	0.660
Divorced	1.58 (0.84 - 2.96)	0.155	1.54 (0.62 - 3.84)	0.355
**HCV knowledge[a], (median, IQR)**	1.06 (0.99 - 1.14)	0.101	1.03 (0.95 - 1.12)	0.432
**Sexual behavior (past 6 months)[c]**				
No sexual contact	1		1	
Unprotected heterosexual contact	1.07 (0.59 - 1.95)	0.820	1.27 (0.63 - 2.57)	0.503
Protected heterosexual contact	1.18 (0.62 - 2.24)	0.619	1.28 (0.58 - 2.82)	0.538
Unprotected same-sex contact	0.71 (0.17 - 3.01)	0.640	0.57 (0.11 - 2.98)	0.508
Protected same-sex contact	1.56 (0.71 - 3.43)	0.264	1.32 (0.43 - 3.99)	0.628
Unprotected bisexual contact	1.86 (0.71 - 4.90)	0.209	1.4 (0.40 - 4.91)	0.603
Protected bisexual contact	1.28 (0.48 - 3.39)	0.621	1.19 (0.34 - 4.15)	0.789
**STI diagnosis (past 6 months)**				

No	1		1	
Yes	1.15 (0.63 - 2.09)	0.657	1.18 (0.63 - 2.21)	0.597
**Sharing needles (past 6 months)**				
No	1		1	
Yes	0.65 (0.35 - 1.21)	0.176	0.73 (0.32 - 1.67)	0.455
**Type of HCV self-test**				
Oral	1		1	
Blood	0.34 (0.21 - 0.56)	<0.001	0.31 (0.19 - 0.52)	<0.001
**Location of HCV self-testing**				
On-site	1		1	
Off-site	4.58 (2.31 - 9.07)	<0.001	3.76 (1.69 - 8.36)	0.001
**Level of assistance needed[b]**				
Unassisted	1		1	
Assisted	0.84 (0.46 - 1.50)	0.547	0.94 (0.50 - 1.78)	0.854

aRR, adjusted risk ratio; ARTC, antiretroviral therapy clinic; CDC, chronic disease clinic; CI, confidence interval; cRR, crude risk ratio; DIC-MSM, drop-in center for men who have sex with men; DIC-PWID, drop-in center for people who inject drugs; HCV, hepatitis C virus; IQR, interquartile range; STI, sexually transmitted infection.

[a]Current HCV knowledge was assessed using 8 questions. Participants who had never heard of HCV were assigned a score of 0, while others received cumulative scores based on correct answers. Scores ranged from 0 to 8, with higher scores indicating greater knowledge, and were treated as a continuous variable.

[b]The variable Level of assistance needed had fewer complete observations (n=2641), which also impacted the total number of observations included in the multivariable analysis.

[c]Sexual behavior categories were constructed by combining reported condom use and the gender of sexual partners in the past 6 months. Participants reporting no sexual contact were classified accordingly. Among sexually active individuals, those who reported 'never' or 'rarely' using condoms were categorized as having unprotected sex, while those reporting condom use 'often' or 'always' were categorized as having protected sex. This composite variable reflects sexual behaviour but not sexual orientation or gender identity.
